# Effect of the Looseness of the Beam End Connection Used for the Pallet Racking Storage Systems, on the Mechanical Behavior of the Bearing Beams

**DOI:** 10.3390/ma15144728

**Published:** 2022-07-06

**Authors:** Florin Dumbrava, Camelia Cerbu

**Affiliations:** 1 Department of Mechanical Engineering, Faculty of Mechanical Engineering, Transilvania University of Brasov, B-dul Eroilor, No. 29, 500036 Brasov, Romania; florin.dumbrava@unitbv.ro; 2 Product Development Department, S.C. Dexion Storage Solutions SRL, Str. Campului Nr. 1A, 505400 Rasnov, Romania

**Keywords:** storage systems, looseness, stiffness, beam-end connection, bending, gap, clearance

## Abstract

The worldwide use of pallet racking storage systems leads to the necessity for research regarding the effects of the clearance between the metallic tabs of the connector and upright slots (looseness effect) on the performance of load-bearing beams. Firstly, the looseness angle and the rotational stiffness were experimentally obtained for three types of beam-to-upright connections. A theoretical approach is used to investigate the magnitude of the looseness effects that occurred on the performances of the bearing beam of the pallet storage systems in terms of the bending moment developed at the midpoint of the beam and maximum deflection. Calculation corrections were evaluated for the connections involved in the experimental part, for the case which considers the looseness effects with respect to the case which does not consider the looseness effect. In order to evaluate the effects of the parameters of the connections on calculus corrections, the theoretical model was used for other types of beam-to-upright connections. It is shown that the maximum corrections are 2.99% and 5.16% for the bending moment developed at the midpoint of the beam and for the maximum deflection, respectively. It is proved that the connector type affects the size of the correction.

## 1. Introduction

Design assisted by testing is a well-known procedure that is often used for design calculus of the pallet storage racking systems. The analysis of the mechanical behavior of semi-rigid connections used on pallet racking systems is generally based on experimental tests according to the European standard EN 15512 [1]. The old version of standard EN 15512 from 2009 [1] was replaced with the updated version, published at the end of 2020 [2]. The procedure for correcting bending moment developed in the bearing beam and deflections caused by the looseness of the beam-end connections was included in the updated version of standard EN 15512-2020 [2]. Looseness effect is not often investigated in steel structure connections. However, this effect has a significant impact on the behavior of the entire structure.

Typical connections used to assembly the uprights with beams, for the racking storage systems, are bolted connections [3,4] and connections with metal tabs [5,6,7,8,9]. Most research on such connections focuses on the analysis of rotational stiffness at ambient temperature [5,6,9] and high temperatures [10]. Bolted connections are a less expensive alternative to tab connectors, used for assembling of the pallet storage systems by taking into account the manufacturing technology of the tab connectors and the upright slots [3]. On the other hand, tab connectors have major advantages in terms of time and flexibility in on-site installation, but also the disadvantage of the initial looseness that should be considered in the design calculus of the racking storage systems having such connectors.

The investigation method regarding the measuring of looseness for beam-upright connections and the looseness effect on the beam deflection and bending moments are shown in the last version of the EN 15512 standard [2]. The experimental investigation of the looseness for beam-to-upright connections could significantly improve the literature in terms of testing the connection elements used in civil engineering, like the ones addressed in a recently published editorial [11].

A looseness effect can also be noticed in the bolted connection. The main difference between connections with metallic tabs and bolted connection is the slippage occurs, in the first case, at the beginning of the moment-rotation curve.

**Figure 1:**
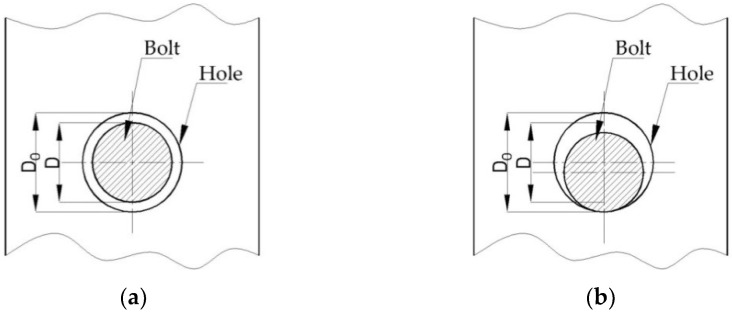
Clearance in bolted connections: (**a**) clearance between bolt and hole; (**b**) bolt in contact with the hole.

Considering the clearance between the bolt and hole (Figure 1), the slip effect has a significant effect on bolted connections in cold formed steel sections [12,13,14]. In the case of the bolted connection, there is a clearance between bolt and hole (Figure 1a), which leads to a sliding effect in the joint after loading. The bolt is subjected to bearing stresses once the slip effect has been overcome. In this phase, the bolt is in contact with the hole, as is shown in Figure 1b.

In literature, there are a lot of scientific works which investigated the behavior of the bolted connections under static or impulsive loads, considering the clearance between bolt and hole [14,15]. By considering the finite element analysis of the models of beam-column connections with bolts, subjected to cyclic loading, Gharebaghi and Hosseini [14] showed that the increasing of the bolt-hole clearance and the decreasing of the pretention force in bolt lead to the reduction of the flexural strength of the bolt connection while the rotation angle increases.

**Figure 2:**
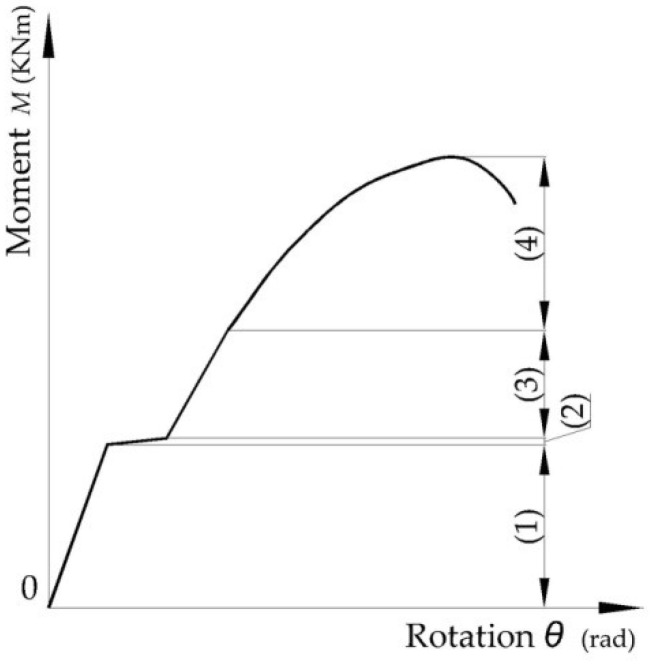
Typical behavior of bolted connections in cold formed steel structures: (**1**) linear behavior before slipping; (**2**) slipping effect caused by clearance between bold and hole; (**3**) linear behavior after cancelling of clearance; (**4**) nonlinear behavior.

The slip effect depends on the clearance between the bold diameter and the hole. In case of the bolted connections, the slip effect happens after a certain load is reached, as shown in Figure 2 for moment-rotation curve recorded for joint with pre-tensioned bolts [13,15,16].

Gilbert and Rasmussen [3] showed that the looseness in bolted connections subjected to bending moment can be neglected in design at ultimate stress state for drive-in and drive-through storage rack systems. Their results are based on the finite element non-linear analysis of the bolted connections between upright and the portal beam for pallets, subjected to cyclic bending moment.

Galeotti et al. [17] showed the positive effects of adding bolts to tab connections for steel storage systems in order to diminish the effect of pinching phenomenon, which appears especially for dynamical loads (earthquake, cyclic loading). The pinching phenomenon affects the looseness angle of the connection.

Godley and Beale [18] showed that the analysis of the scaffold structures must consider non-linear joint models since the moment-rotation curve is usually not the same for positive and negative rotation and the looseness appears. The looseness influences the overall deflection of the scaffold structure but its effects on the load-bearing capacity for the braced frames may be neglected [18,19]. Prabhakaran et al. [19] showed a method for the non-linear analysis of the scaffold structures, taking into account the looseness effects in the joints.

Contrary to the bolted connections, the European standard EN 15512 [2] and scientific work [3] require considering the looseness of the tab connectors used between the upright and beam in stability analysis of the storage racking systems in the down-aisle direction. The down-aisle represents the direction parallel to a run.

**Figure 3:**
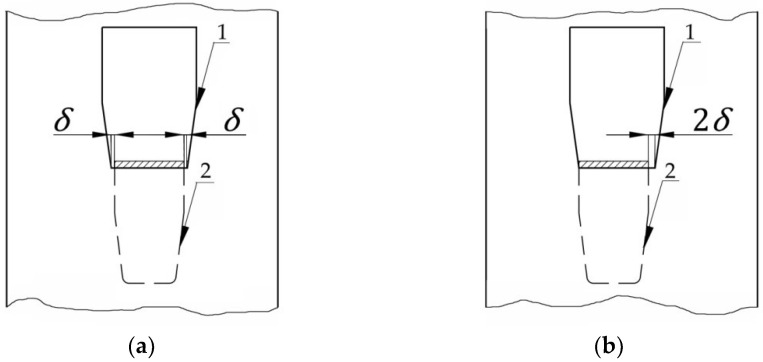
Looseness effect of beam end connector: (**a**) clearance between tab of the beam-end connector and upright slot; (**b**) tab of the connector will is in contact with one side of the upright slot. Note: (**1**) slot of the upright; (**2**) tab of the beam-end connector; *δ* is clearance.

On the other hand, the slip effect (looseness angle, as is mentioned in EN15512) happened at a lower load in case of the tab connections compared with the bolted connections [2]. This can be very simply explained because the bolts are generally pre-tensioned. In the case of the tab connections which are designed for the pallet racking systems, it is difficult to establish a general rule to take in consideration the looseness effect because of different shapes of both the metallic tabs and upright’s slots. For these reasons, the agreed procedure is based on experimental tests. The difference between the metal tab width and the upright slot dimensions has a direct impact on the looseness *δ* (Figure 3). Until the metallic tab will not be in contact with the upright’s slot (Figure 3a), the connection will behave like a pinned connection. Once the tab gets in contact with the upright slot (Figure 3b), the connection behaves like a semi-rigid connection.

There are some published papers [5,6,9] which approach the experimental testing of the beam-to-upright end connections for steel storage system, with tab connectors, under bending loading, without considering the looseness effects. Investigations conducted on the rotation stiffness of the tab connections showed that the increasing of the height of the connector or the increasing of the number of the tabs of the connector, respectively, lead to the increasing of the connection rigidity [6,20], while the effect of the gap between the beam and the face of the tab connector could be neglected [20].

Moreover, Escanio [9] validated the numerical models of such a beam-to-upright end connection subjected to bending, by correlation with the experimental results obtained for different combination between three types of uprights and four kinds of beams whose cross sections were different. However, the looseness between the metallic tabs and upright slots was not approached in the experimental program.

In literature, there are also analytical methods used to estimate the rotational stiffness and bending capacity for a rack connection with metallic tabs. The component method represents a good alternative to the experimental tests. Considering the component method, Gusella et al. [8] showed how the connections for racking storage systems are affected by the uncertainty in steel mechanical properties and geometrical features. In another research [21], the values for the initial rotational stiffness of beam-to-upright connections were also evaluated based on component method and compared with the experimental results. In that research, Zhao et al. [21] concluded that the maximum ratio between the theoretical and the experimental results was equal to 1.44 for the beam-to-upright connections involved in their research.

In this context, the accuracy predicting of the mechanical behavior of the tabs connection subjected to bending loading, including the looseness effect, represents an important aspect for the structural behavior of the beam-connector-upright assembly, especially for pallet racking systems. The experimental findings on the rotational stiffness and on looseness of the beam-to-upright connections used for storage racking systems can be used in numerical analysis for prediction of the mechanical behavior of the entire storage structure [22].

The main purpose of this research is to investigate the looseness that occurs in the tab connector in beam-upright connections and to evaluate the influence of looseness effect for the portal beam loaded with pallets, having different types of tab connectors at the beam-ends. For this purpose, the main objectives of this research are: (i) experimental determination of the looseness for three types of beam-connector-upright assemblies involving three types of uprights (different thickness of the section wall); (ii) considering the looseness effect occurring for the tab connections, in an analytical model used for computing of the bending moment developed at the middle of the beam and maximum deflection of the beam; (iii) comparison regarding to the looseness effect on the bending moment and maximum deflection, for the portal beam loaded with pallets, having different configurations for the beam-end connections.

## 2. Materials and Methods

### 2.1. Assemblies Subjected to Looseness Tests

The purpose of the experimental program is to determine the looseness angle for three different beam-to-upright connections, which are presented in Table 1. The corresponding identification codes, which are referred to further in this research, are also shown in Table 1.

**Figure 4:**
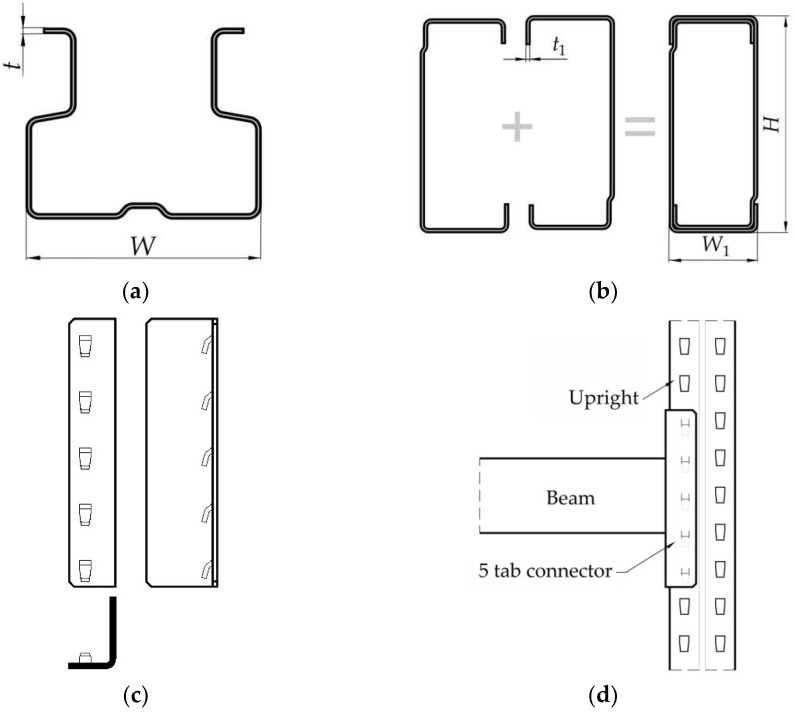
Elements of the upright-connector-beam assemblies tested: (**a**) upright sections; (**b**) beam section; (**c**) connector with 5 tabs; (**d**) sketch of the tested assembly.

The elements of the upright-connector-beam assemblies tested are shown in Figure 4. Three different upright sections (Figure 4a) are used, which have the same shape for cross section, but three different wall thicknesses (1.50 mm, 1.75 mm, and 2.00 mm), as shown in Table 1. The same boxed beam, which is obtained from two C-profiles brought together (Figure 4b), is connected with the upright with a five tab connector (Figure 4c) that is welded at the end of the beam. For every tested assembly (Figure 4d and Table 1), the same type of five tabs connector was used, denoted as 5T in Table 1. It was used a beam having a large second moment of area with respect to the major axis in order to reduce, as much as possible, the bending effect of the beam in the mechanical tests made for obtaining of the looseness angle.

Test assembly is presented in Figure 4d. A set of four assemblies was prepared for every looseness test setup (penultimate column of Table 1), resulting in total 12 individual tests. A set of six beam-connector-upright assemblies (last column of Table 1) was prepared for each bending test to determine the rotational stiffness.

### 2.2. Work Method for Bending and Looseness Tests on Beam End Connectors

Three different beam-connector-upright assemblies were investigated in the bending test and then for the looseness angle, as shown in Table 1, in accordance with EN 15512 standard [2].

**Figure 5:**
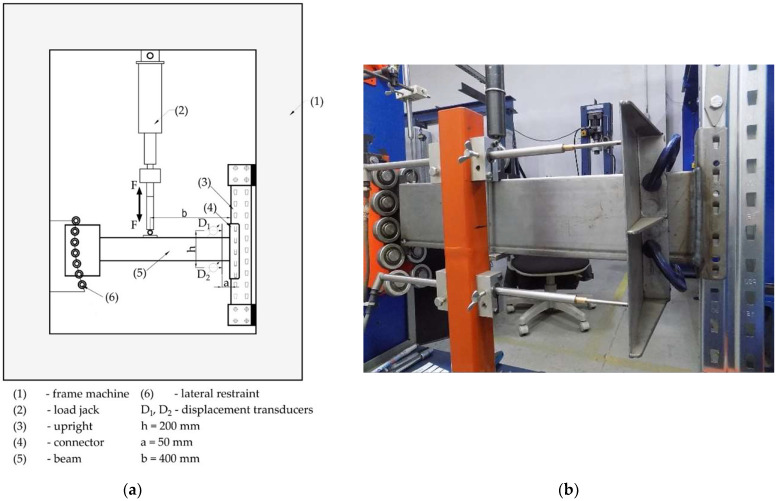
Experimental test setup for bending test and looseness test according to European standard EN 15512: (**a**) scheme of the test stand; (**b**) photo of the test stand.

The experimental test setup is presented in Figure 5 and it is the same for the both bending test and looseness test for the beam end connectors, according to European standard EN 15512 [2]. The scheme of loading is shown in Figure 5a, while a photo of the test stand is given in Figure 5b. Firstly, the bending test of each type of beam-connector-upright assembly was made in order to determine the design moment denoted with *M_Rd_* and the rotational stiffness *k_m_*. The bending test for the beam end connectors, and the method for obtaining both the design moment *M_Rd_* and the rotational stiffness *k_m_*, are described in detail by the authors of this research in their previously published research [6], according to European standard EN 15512 [2]. For the looseness test, it is mentioned that the loading jack must be capable of applying both downward force and upward force (Figure 5a) in order to apply bending moment in reverse direction.

A rigid plate is fixed on the beam, on which the displacement transducers are bearing, to measure the rotation angle of the beam in connection by using the test stand (Figure 5b). In order to avoid the influence of the connector distortion on the rotation angle measured, the distance of 50 mm (dimension a in Figure 5) is set between the connection and the plate fixed on the beam, on which the displacement transducers are bearing, used to measure the rotation angle. This clearance between the connection and the plate fixed on the beam was established according to European standard EN 15512 [2] and the test method was described in other research [23,24,25]. Prabha et al. [26] used an inclinometer fixed on beam, close to the connection, in order to measure the rotation angle of the beam for evaluation of the stiffness of the connection. It also avoids the effects of the local deformations of the beam which occurred near the connector during the cantilever test. On the other hand, the distance between connector and plate fixed on the beam is required because there is the weld between beam and connector.

For both the bending test and looseness test, the moment-rotation (M−θ) curve is required to be plotted. Considering the notations shown in Figure 5a, the bending moment *M* developed at beam end connector and the rotation angle *θ* of the connector, expressed in radians, are computed by using Equation (1) and Equation (2), respectively [2].

(1) M=bF,

(2) $$\theta =\left(D_{1}-D_{2}\right)/h.$$

In the looseness test, the force *F* (Figure 5a) was slowly increased until the moment the connector reached a value of 10% of the design moment denoted with *M_Rd_*. Further, the load was gradually reduced and then reversed in the opposite direction, until the moment reached 10% of the design moment *M_Rd_*, in that direction.

**Figure 6:**
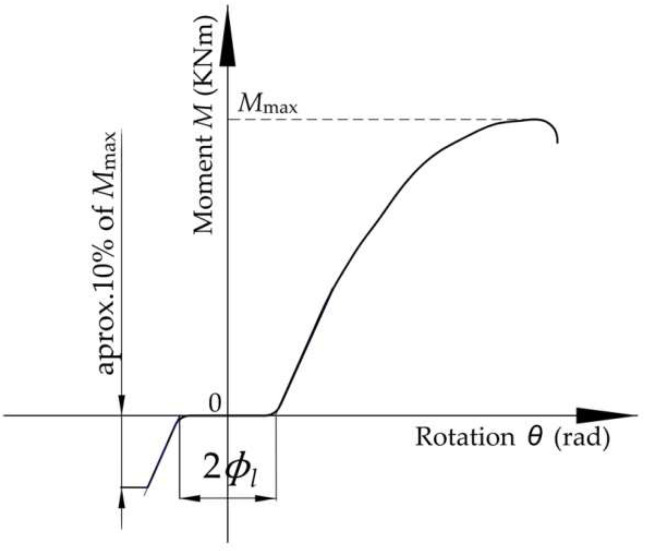
Typical moment rotational curve as test output to determine the looseness angle $\Phi_{l}$.

Twice the looseness angle denoted with $2\Phi_{l}$ was measured for each looseness test by extrapolating the linear parts of the moment-rotation curve towards the origin to find their intersection points with the rotation axis, as shown in Figure 6. The difference between the abscissas corresponding to the two points of intersection obtained in this manner is equal to twice the looseness angle $\Phi_{l}$ of the beam end connector.

All moment rotation (M−θ) curves obtained for the beam-connector-upright assemblies involved in this research are reported as results. The paper also reports the following data obtained by processing of the experimental data: looseness angle $\Phi_{l}$, design moment *M_Rd_*, and rotational stiffness *k_m_*.

### 2.3. Theoretical Approach

**Figure 7:**
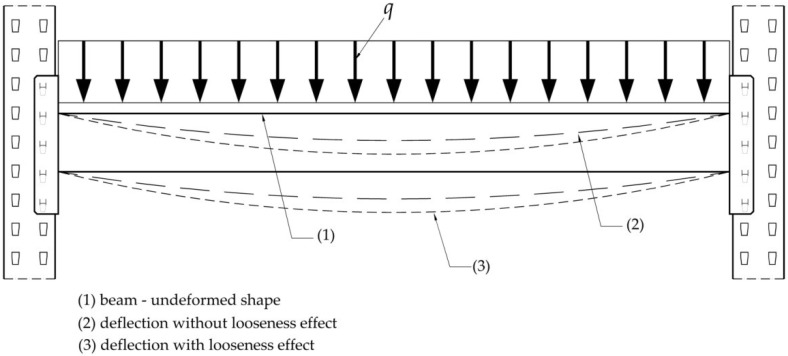
Looseness effect on the deflection of typical beam of the storage racking systems.

In fact, the looseness effect is caused by the gap between the width of the metallic tabs of the connector and the width of the slots located on the upright front side. In Figure 7, it is shown how the effect of looseness affects the real deflection of the beams in operation on storage systems. The beam shape before deformation is drawn with a continuous line. After loading of the beams with pallets, the deformed shape of the beam is drawn with a dashed line without considering the looseness effect, and it is also drawn with a dotted line by taking into account the looseness effect (Figure 7).

It has to be mentioned that the simplified static scheme shown in Figure 7 is considered for the portal beam, in accordance with the Annex C of the European standard EN 15512: 2020 [2], in order to make correction of bending moment and deflection due to looseness. In the design of the pallet racking storage systems, it is usually considered that the distributed force *q*, acting on the beam shown in Figure 7, is caused by the weight of the pallets supported by the beams of a shelf.

**Figure 8:**
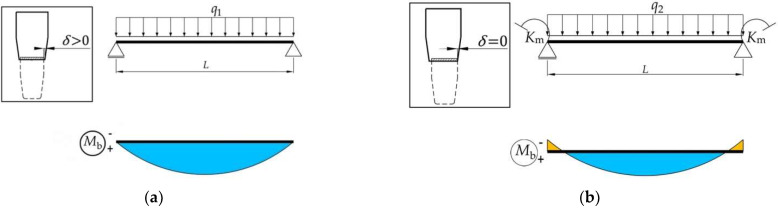
Beam design by considering the looseness effect of beam end connector: (**a**) the first step (pinned connections); (**b**) the second step (semi-rigid connections).

According to Annex C of the European standard EN 15512: 2020 [2], in operation of the tab connections, the rotational stiffness of the connection *k_m_*, which is determined by experimental tests, works once the looseness angle $\Phi_{l}$ is reached. Until this value rotation angle, the connection behaves like a pin-connection. Therefore, theoretical approach to include the looseness effect in the structural design is divided in two steps in Figure 8: the first step is presented in Figure 8a, for which the tab connection behaves like a pin-connection; the second step is presented in Figure 8b, when the tab connection behaves like a semi-rigid connection whose rotational stiffness *k_m_* is experimentally determined.

In this manner, the European standard EN 15512: 2020 [2] neglects that the rotational stiffness *k_m_* and the looseness angle $\Phi_{l}$ are experimentally determined in bending test of the beam-connector-upright assembly, when the beam was subjected to the concentrated force applied to its free end, for which the work method is described in Section 2.2 of this research.

It is well-known from literature that the rotation *φ* of the beam end of the simply supported beam subjected to the uniformly distributed force *q* is computed by using Equation (3) [27].

(3) $$\varphi =qL^{3}/\left(24EI\right),$$

where E is the modulus of elasticity of the material of the beam; I is the second moment of inertia of the beam cross section with respect to the neutral axis; EI represents stiffness modulus in bending that is constant along the axis of the beam, having the length L.

From Equation (3), it may compute the distributed force $q_{1}$ (Figure 8a) that is needed to cover the looseness angle $\Phi_{l}$ by Equation (4).

(4) $$q_{1}=24EI\Phi_{l}/L^{3}.$$

The distributed force $q_{1}$, acting on simply supported beam (Figure 8a), causes the bending moment $M_{mid,\;\Phi_{l}}$ developed at the middle of the beam and the maximum deflection $v_{max,\;\Phi_{l}}$ of the middle of the beam, which are computed by Equation (5) and Equation (6), respectively [27].

(5) $$M_{mid,\Phi_{l}}=q_{1}L^{2}/8.$$

(6) $$v_{max,\Phi_{l}}=5q_{1}L^{4}/\left(384EI\right).$$

Once the looseness angle $\Phi_{l}$ has been reached, the connection behaves like a semi-rigid connection. From this point further, assuming that the weight of the pallets is placed on the same shelf and is uniformly distributed, the corresponding distributed force *q* is computed by Equation (7) according to European standard 15512 [2].

(7) $$q=\frac{n_{p}F_{p}\gamma_{F}}{2L},$$

where $n_{p}$ is the number of pallets; $F_{p}$ is the weight of one pallet; $\gamma_{F}$ is the load factor that is equal to 1.40 [2].

The uniformly distributed force $q_{2}$, which is applied on the beam length *L* in the second case of loading after the looseness angle $\Phi_{l}$ is covered (Figure 8b), is computed by subtracting the load $q_{1}$ from the load q by Equation (8).

(8) $$q_{2}=q-q_{1}=n_{p}F_{p}\gamma_{F}/\left(2L\right)-24EI\Phi_{l}/L^{3}.$$

For the beam shown in Figure 8b, the rotational stiffness of the both end connections is denoted with $k_{m}$ and it is experimentally investigated according to European standard 15512 [2].

The bending moment $M_{end,\;q_{2}}$ developed at the both beam end connections and the bending moment $M_{mid,\;q_{2}}$ developed at the middle of the beam, shown in Figure 8b, are computed by Equation (9) and Equation (10), respectively, according to the recently published paper [6] by the authors of the present research.

(9) $$M_{end,\;q2}=\frac{q_{2}L^{3}}{24EI\left(\frac{1}{k_{m}}+\frac{L}{2EI}\right)}.$$

(10) $$M_{mid,q2}=q_{2}L^{2}/8-M_{end,q2}.$$

The maximum deflection $v_{max,\;q_{2}}$ of the middle of the beam shown in Figure 8b is computed by using Equation (11), given by the authors in the recently published paper [6].

(11) $$v_{max,\;q_{2}}=\frac{q_{2}L^{4}\left(10EI+Lk_{m}\right)}{384EI\left(2EI+Lk_{m}\right)}.$$

Using the method superposition of effects, the total bending moment $M_{mid,\;t}$ developed at the middle of the beam and the maximum deflection $v_{max,\;t}$ of the middle of the beam are computed with Equation (12) and Equation (13), respectively.

(12) $$M_{mid,t}=M_{mid,\Phi_{l}}+M_{mid,q2}.$$

(13) $$v_{max,\;t}=v_{max,\Phi_{l}}+v_{max,\;q_{2}}.$$

It is noted that both Equations (12) and (13) take into account the looseness effects of the beam-upright connectors located at both beam ends.

If the looseness effects of the beam-upright connectors are neglected, replacing the distributed force $q_{2}$ with q in Equations (10) and (11) leads to the mathematical expressions of the bending moment $M_{mid}$ developed at the middle of the beam and of the maximum deflection $v_{max}$, given by Equation (14) and Equation (15), respectively.

(14) $$M_{mid}=qL^{2}/8-\frac{qL^{3}}{24EI\left(\frac{1}{k_{m}}+\frac{L}{2EI}\right)}.$$

(15) $$v_{max}=\frac{qL^{4}\left(10EI+Lk_{m}\right)}{384EI\left(2EI+Lk_{m}\right)}.$$

Finally, the calculation corrections (denoted with *CORR*) concerning the bending moment and maximum deflection considering the looseness effect are computed by Equations (16) and (17), with respect to the case of the beam for which the looseness effects of the beam-upright connectors are neglected.

(16) $$CORR_{M_{mid}}=\frac{\left|M_{mid,\;t}-M_{mid}\right|}{M_{mid}}\cdot 100\;\left(\%\right).$$

(17) $$CORR_{v_{max}}=\frac{\left|v_{max,\;t}-v_{max}\right|}{v_{max}}\cdot 100\;\left(\%\right).$$

In order to comparatively analyze the looseness effects on the beam deflection for different combinations of beams, uprights, and connectors, the experimental results shown in Table 2 are additionally considered, reported by the authors in the previously published research article [6] regarding the rotational stiffness $k_{m}$ and design moment $M_{Rd}$ obtained for other beam-connector-upright assemblies, which are similar to the ones involved in this research. The bending moment developed at the middle of the beam and maximum deflection of the beam are computed by using the Equation (12) and Equation (13), respectively, considering the looseness effects occurred at the connectors with tabs located at both beam ends. The results are compared with the ones obtained by using Equations (14) and (15), which are valid when the looseness effects that occurred at tab connections are neglected.

## 3. Results and Discussion

### 3.1. Experimental Results

**Figure 9:**
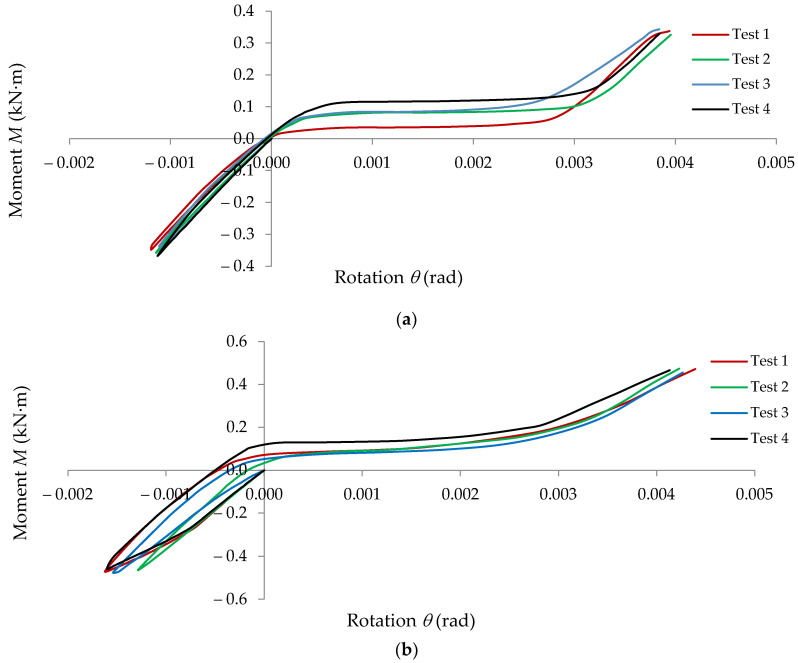

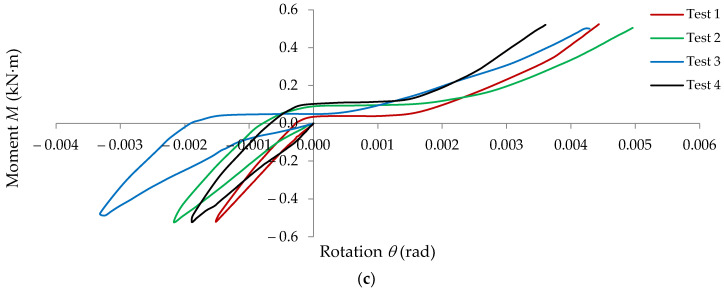
Moment-rotation (*M* − *θ*) curves recorded in looseness tests for the beam-connector-upright assemblies: (**a**) 0-I-5L; (**b**) 0-II-5L; (**c**) 0-III-5L.

In Figure 9, the moment-rotation (*M* − *θ*) curves recorded in looseness tests are shown for the following beam-connector-upright assemblies: 0-I-5L; 0-II-5L; 0-III-5L.

**Figure 10:**
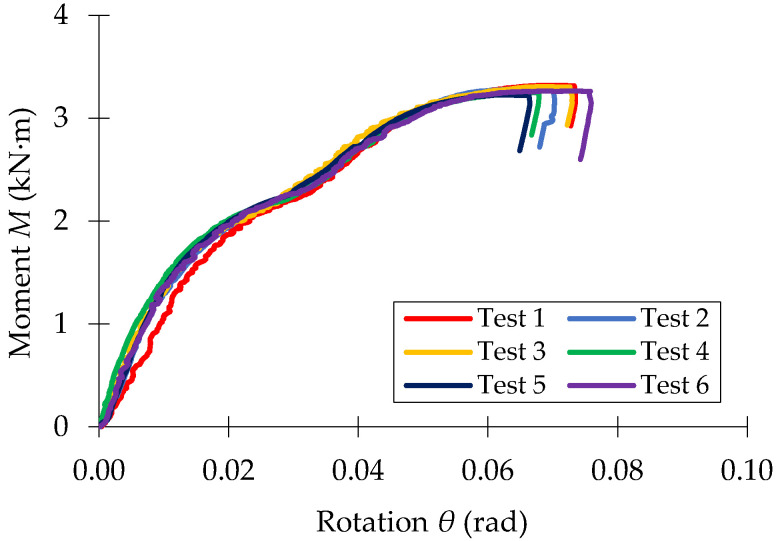
Moment-rotation curve (M−θ) recorded in bending tests for the beam-connector-upright assembly of type 0-I-5T.

**Figure 11:**
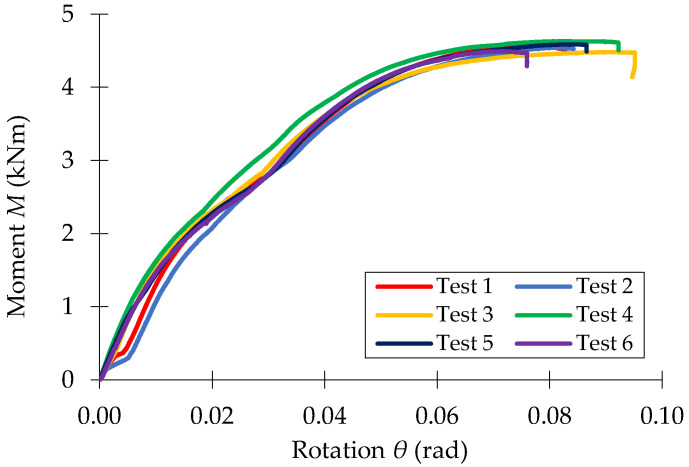
Moment-rotation curve (M−θ) recorded in bending tests for the beam-connector-upright assembly of type 0-II-5T.

**Figure 12:**
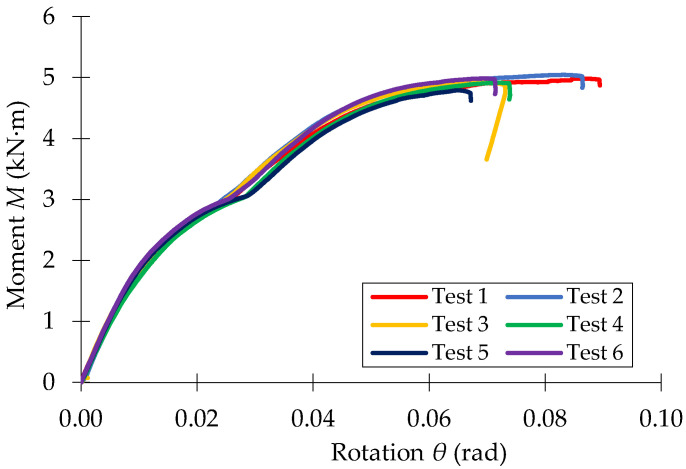
Moment-rotation curve (M−θ) recorded in bending tests for the beam-connector-upright assembly of type 0-III-5T.

The moment-rotations (*M* − *θ*) curves recorded in bending tests of the three beam-connector-upright assemblies investigated (0-I-5L, 0-II-5L, 0-III-5L) are shown in Figure 10, Figure 11 and Figure 12, respectively.

The experimental results regarding the looseness angle $\Phi_{l}$, design moment $M_{Rd}$, and rotational stiffness $k_{m}$ corresponding to the beam-connector-upright assemblies involved in this research are summarized in Table 3.

### 3.2. Effects of Looseness of the Semi-Rigid Connections on Mechanical Behavior of the Beam

In order to evaluate the effects of the looseness which takes place at beam-to-upright connections with tabs, it is necessary to investigate the maximum deflection and bending moment developed for the bearing beam being in operation on the storage pallet racking systems. It is considered that the shelf of the storage system consists of two beams having the length of 2.7 m, which must support the maximum weight of 15,000 N corresponding for loading with three pallets. Assuming that the load of 7500 N corresponding to one beam of the shelf is uniformly distributed on the beam length of 2.7 m, the scheme of loading of the bearing beam shows like that shown in Figure 7, subjected to the uniformly distributed force q of 2.78 N/mm (7500 N distributed over 2.7 m).

The looseness effects are investigated for all types of beam-to-upright connections, whose values for the rotational stiffness $k_{m}$ are given in Table 2 and Table 3. Because the looseness angle $\Phi_{l}$ depends mainly on the combination between upright and tabs connectors, it is assumed that the looseness angle $\Phi_{l}$ remains the same for all beam-connector-upright assemblies that combine the same type of upright (type I, II, or III) and connector with 4 tabs (denoted with 4T) or 5 tabs (denoted with 5T). The critical case of the beam-to-upright connections with 5 metallic tabs, denoted with 5T, was involved in looseness tests in this research (Table 1). Taking into account this assumption, the results given in Table 3 for the looseness angle $\Phi_{l}$ were extended for the beam-connector-upright assemblies shown in Table 2. The values of the looseness angle $\Phi_{l}$, considered in calculus for each connection type, are given in the second column of Table 4.

For the study case considered above, consisting of a beam whose length *L* is 2.7 m, subjected to the uniformly distributed force *q* of 2.78 N/mm, having the same beam-to-upright connection at its both ends, the bending moment developed at the middle of the beam and the maximum deflection were computed with Equation (12) and Equation (13), respectively, considering the looseness effects occurred at both beam-end connectors. The results obtained for all beam-connector-upright assemblies are summarized in Table 4. Additionally, the same quantities (bending moment at midpoint of the beam and maximum deflection) were computed for all beam-connector-upright assemblies involved without considering the looseness effect, and the results are also given in Table 4.

To highlight the looseness effects, the calculation corrections concerning both the bending moment $M_{mid}$ developed at the middle of the beam and the maximum deflection $v_{max}$ of the beam are computed with Equation (16) and Equation (17), respectively, with respect to the case of the beam for which the looseness effects of the beam-upright connectors are neglected. The results regarding the calculation corrections are summarized in the last two columns of Table 4. It is remarked that the maximum corrections for both the bending moment developed at the midpoint of the beam and the maximum deflection are recorded for the beam-connector-upright assembly, having the code 0-II-5T.

## 4. Discussion

**Figure 13:**
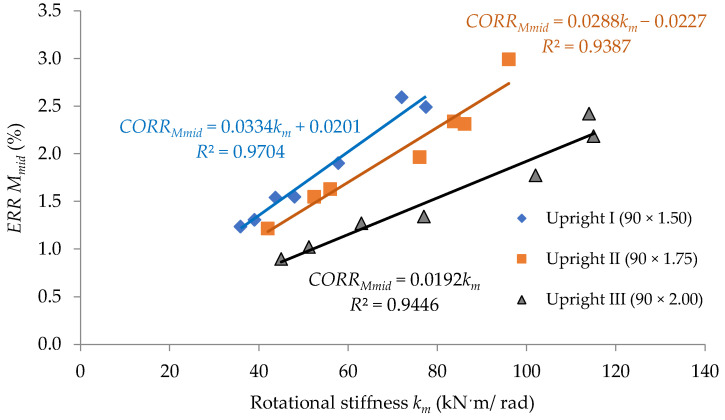
Variation of the calculation correction concerning the bending moment developed at midpoint of the beam related to the rotational stiffness *k_m_* of the connection for each type of the upright involved.

**Figure 14:**
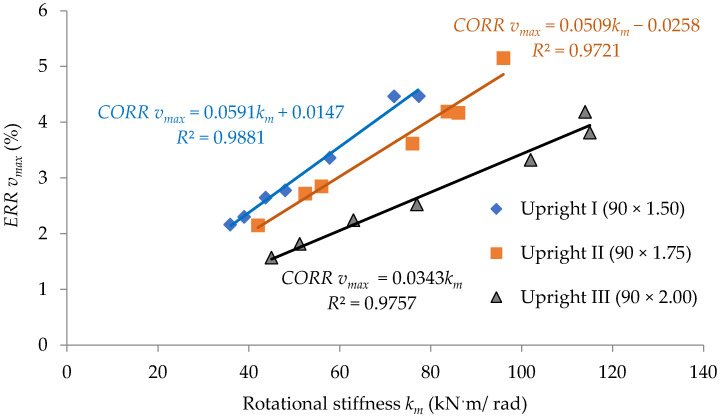
Variation of the calculation correction concerning the maximum deflection of the beam related to the rotational stiffness *k_m_* of the connection for each type of the upright involved.

In order to interpret the effect of the looseness that occurred at beam-to-upright connections, on the size of the corrections concerning the calculus of both the bending moment $M_{mid}$ developed at midpoint of the beam and the maximum deflection $v_{max}$ of the beam, with respect to the type of the upright and with respect to the rotational stiffness $k_{m}$, it is used for the plots shown in Figure 13 and Figure 14.

In Figure 13 and Figure 14, the results regarding the corrections are approximated by using linear functions with respect to the rotational stiffness, $k_{m}$, for each type of upright involved in this study. The least squares method was used for approximation of data in both Figure 13 and Figure 14, where the value *R*^2^ close to 1 shows that the data are accuracy approximated. It is observed that for close values of the rotational stiffness $k_{m}$, the correction is even greater the thinner the wall thickness of the upright. Those functions shown in Figure 14 could be used in further research to evaluate the size of the corrections, which should be considered in calculation of the maximum deflection $v_{max}$ by considering the looseness effect for a certain type of beam-to-upright connection with tabs. Of course, the approximation functions shown in Figure 13 and Figure 14 only cover the beam-to-upright connections involved in this study.

**Figure 15:**
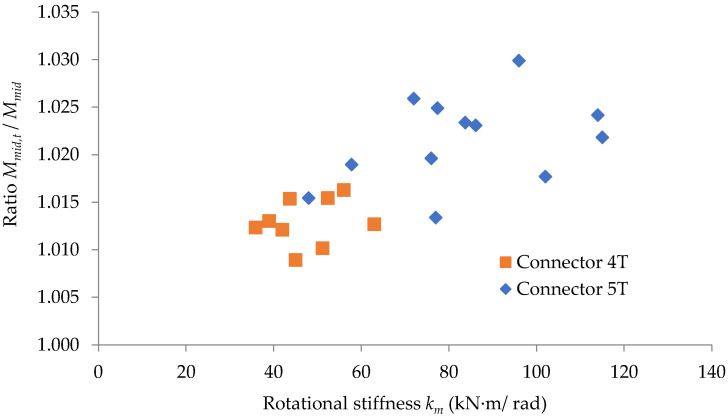
Variation of the ratio $M_{mid,\;t}/M_{mid}$ related to the rotational stiffness *k_m_* of the connection for each type of tabs connector involved.

In Figure 15, the ratio between the bending moment developed at the midpoint of the beam computed by considering the looseness effect of the connection and the same quantity computed without considering that effect is plotted with respect to the rotational stiffness $k_{m}$, with two different colors to highlight the effect of the type of the connector. It may be observed that this ratio is greater for the connector 5T, having five tabs compared to the connector 4T with four tabs.

**Figure 16:**
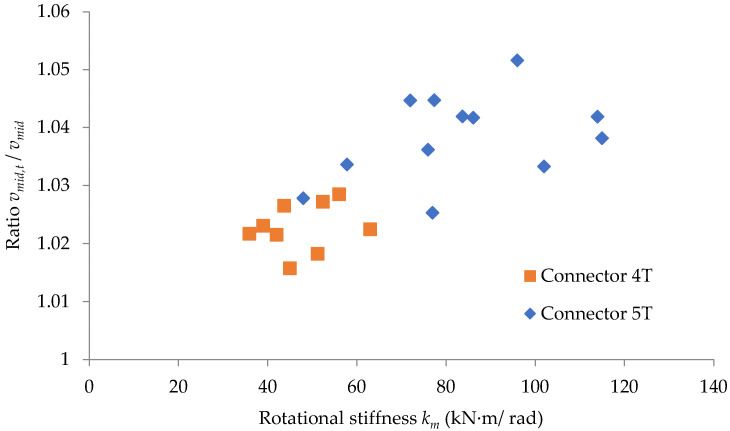
Variation of the ratio $v_{max,\;t}/v_{max}$ related to the rotational stiffness *k_m_* of the connection for each type of tabs connector involved.

In a similar way, in the plot shown in Figure 16, it is remarked that the ratio between the maximum deflections computed by considering and without considering the looseness effect is in the range 1.016–1.029 for the connector 4T (with four tabs), while this ratio is in the range 1.025–1.052 for the connector 5T (with five tabs).

The reliability in results concerning the looseness effects could be affected by the magnitude of the vertical load applied to the beam in operation of the storage racking system. Plastic deformation of the tabs of the connector, caused by dynamical loads (earthquake, cyclic loading) applied in the past, could also influence the looseness angle of the tabs connection. Jovanovic et al. [28] have already shown that cyclic loading applied to the beam-to-upright connection of the steel racking systems lead to the increasing of the looseness angle. As a result, the looseness effects on both the deflection of the beam and strength capacity of the beam are much more pronounced in the case of dynamic loading.

## 5. Conclusions

This paper presents the results experimentally obtained for the looseness angle $\Phi_{l}$ according to the methodology of the European standard 15512 [2], recently updated, for different beam-connector-upright assemblies. Moreover, the effects of the looseness that takes place in connection are evaluated and interpreted concerning the values of the maximum deflection of the bearing beam of the racking pallets system and considering the bending moment developed at the midpoint of that beam.

It may be concluded that there are maximum calculation corrections of 2.99% and 5.16% for the bending moment developed at the midpoint of the beam and for the maximum deflection, respectively, computed by considering the looseness effect with respect to the same quantities computed without considering the looseness effect.

The graphic interpretation of the results proves that the corrections regarding both the bending moment developed at the midpoint of the beam and the maximum deflection are all the more significant the thinner the upright wall. It is also remarked that the ratio between the maximum deflections, computed by considering and without considering the looseness effect, is greater for the connector 5T than for the connector 4T.

In practice, the design engineers should take into account the looseness effects on the maximum deflection of the bearing beam of the racking pallets systems and also on the bending moment developed at the midpoint of the beam, especially for values greater than 80 kN·m/rad of the rotational stiffness of the connection.

## Figures and Tables

**Table 1:** Upright-connector-beam assemblies tested.

Assembly Code	Upright *W* * × *t* *	Beam *H* ** × *W*_1_ ** × *t*_1_ **	Connector Type	Number of Looseness Tests for Assembly	Number of Bending Tests for Assembly
0-I-5T	90 × 1.50	BOX 150 × 50 × 1.75	5T	4	6
0-II-5T	90 × 1.75			4	6
0-III-5T	90 × 2.00			4	6

* Dimensions of the uprights are shown in Figure 4a. ** Dimensions of the beam are shown in Figure 4b.

**Table 2:** Characteristics of the upright-connector-beam assemblies involved in analysis of the looseness effect on beam’s deflection and bending moment developed, adapted from ref. [6].

Assembly Identification Code	Upright *W* * × *t* *	Beam (*H* ** × *W*_1_ ** × *t*_1_ **)	Connector Type	Rotational Stiffness $k_{m}$ (kN·m/rad)	Design Moment $M_{Rd}$ (kN·m)
A-I-4T	90 × 1.50	A (BOX 90 × 40 × 1.25)	4T	39	1.54
A-I-5T			5T	48	2.30
A-II-4T	90 × 1.75		4T	42	2.43
A-II-5T			5T	76	2.21
A-III-4T	90 × 2.00		4T	45	2.16
A-III-5T			5T	77	2.09
B-I-4T	90 × 1.50	B (BOX 100 × 40 × 1.25)	4T	35.9	1.38
B-I-5T			5T	57.8	2.24
B-II-4T	90 × 1.75		4T	56	2.03
B-II-5T			5T	86.1	2.36
B-III-4T	90 × 2.00		4T	51.2	2.43
B-III-5T			5T	102	2.18
C-I-4T	90 × 1.50	C (BOX 110 × 40 × 1.25)	4T	43.7	1.70
C-I-5T			5T	77.4	2.15
C-II-4T	90 × 1.75		4T	52.4	2.74
C-II-5T			5T	83.7	2.95
C-III-4T	90 × 2.00		4T	63	2.89
C-III-5T			5T	115	2.61

* Dimensions of the uprights are shown in Figure 4a. ** Dimensions of the beam are shown in Figure 4b.

**Table 3:** Results obtained in looseness tests and bending tests for all assemblies involved in the experimental program.

Assembly Code	Looseness Angle			Design Moment		Rotational Stiffness	
	$\Phi_{l\;i}$ (rad)	$\Phi_{l}$ (rad)	Stdev (rad)	$M_{Rd}$ (kN·m)	Stdev (kN·m)	$k_{m}$ (kN·m/rad)	Stdev (kN·m/rad)
0-I-5T	0.00139	0.00133	0.000124	2.91	0.035	72	10.029
	0.00140						
	0.00114						
	0.00137						
0-II-5T	0.00115	0.00116	0.000171	4.02	0.058	96	4.118
	0.00126						
	0.0013						
	0.00092						
0-III-5T	0.00075	0.00080	0.000104	4.32	0.086	114	4.425
	0.00093						
	0.00083						
	0.00069						

**Table 4:** The looseness effects on the bending moment and maximum deflection at the middle of the beam for the upright-connector-beam assemblies involved.

Assembly Code	Looseness Angle $\Phi_{l}$ (rad)	With Looseness Effects		Without Looseness Effects		$CORR_{M_{mid}}$ (%)	$CORR_{v_{max}}$ (%)
		Bending Moment at Mid. $M_{mid,\;t}$ (kN·m)	Max. Deflection at Mid. $v_{max,\;t}$ (mm)	Bending Moment at Mid. $M_{mid}$ (kN·m)	Max. Deflection at Mid. $v_{max}$ (mm)		
0-I-5T	0.00133	3.245	2.83	3.163	2.709	2.59	4.47
0-II-5T	0.00116	3.168	2.753	3.076	2.618	2.99	5.16
0-III-5T	0.00080	3.089	2.662	3.016	2.555	2.42	4.19
A-I-4T	0.00133	2.794	12.383	2.758	12.104	1.31	2.31
A-I-5T		2.692	11.831	2.651	11.511	1.55	2.78
A-II-4T	0.00116	2.753	12.153	2.72	11.897	1.21	2.15
A-II-5T		2.441	10.45	2.394	10.085	1.96	3.62
A-III-4T	0.00080	2.709	11.884	2.685	11.7	0.89	1.57
A-III-5T		2.419	10.297	2.387	10.043	1.34	2.53
B-I-4T	0.00133	2.947	10.325	2.911	10.106	1.24	2.17
B-I-5T		2.735	9.429	2.684	9.122	1.90	3.37
B-II-4T	0.00116	2.744	9.455	2.7	9.193	1.63	2.85
B-II-5T		2.522	8.515	2.465	8.174	2.31	4.17
B-III-4T	0.00080	2.774	9.561	2.746	9.39	1.02	1.82
B-III-5T		2.41	8.01	2.368	7.752	1.77	3.33
C-I-4T	0.00133	2.968	8.321	2.923	8.106	1.54	2.65
C-I-5T		2.715	7.478	2.649	7.158	2.49	4.47
C-II-4T	0.00116	2.887	8.043	2.843	7.83	1.55	2.72
C-II-5T		2.668	7.306	2.607	7.012	2.34	4.19
C-III-4T	0.00080	2.79	7.693	2.755	7.524	1.27	2.25
C-III-5T		2.479	6.64	2.426	6.396	2.18	3.81

## Data Availability

Not applicable.
